# Costs and Quality of Life in Diabetic Macular Edema: Canadian Burden of Diabetic Macular Edema Observational Study (C-REALITY)

**DOI:** 10.1155/2014/939315

**Published:** 2014-03-26

**Authors:** John R. Gonder, Valery M. Walker, Martin Barbeau, Nancy Zaour, Bryan H. Zachau, James R. Hartje, Ruihong Li

**Affiliations:** ^1^Ivey Eye Institute, St. Joseph's Health Centre, 268 Grosvenor Street, London, ON, Canada N6A 4V2; ^2^OptumInsight, 5500 North Service Road, Suite 501, Burlington, ON, Canada L7L 6W6; ^3^Novartis Pharmaceuticals Canada, 385 Bouchard Boulevard, Dorval, QC, Canada H9S 1A9

## Abstract

*Purpose*. To characterize the economic and quality of life burden of diabetic macular edema (DME) in Canadian patients. *Patients and Methods*. 145 patients with DME were followed for 6 months with monthly telephone interviews and medical chart reviews at months 0, 3, and 6. Visual acuity in the worst-seeing eye was assessed at months 0 and 6. DME-related healthcare costs were determined over 6 months, and vision-related (National Eye Institute Visual Functioning Questionnaire) and generic (EQ-5D) quality of life was assessed at months 0, 3, and 6. *Results*. Mean age of patients was 63.7 years: 52% were male and 72% had bilateral DME. At baseline, visual acuity was categorized as normal/mild loss for 63.4% of patients, moderate loss for 10.4%, and severe loss/nearly blind for 26.2%. Mean 6-month DME-related costs/patient were as follows: all patients (*n* = 135), $2,092; normal/mild loss (*n* = 88), $1,776; moderate loss (*n* = 13), $1,845; and severe loss/nearly blind (*n* = 34), $3,007. Composite scores for vision-related quality of life declined with increasing visual acuity loss; generic quality of life scores were highest for moderate loss and lowest for severe loss/nearly blind. *Conclusions*. DME-related costs in the Canadian healthcare system are substantial. Costs increased and vision-related quality of life declined with increasing visual acuity severity.

## 1. Introduction

Diabetic macular edema (DME) is a complication of diabetic retinopathy and a major cause of visual impairment and blindness in patients with diabetes [1, 2]. Increased vascular permeability results in the accumulation of extracellular fluids in the retina, leading to retinal thickening and loss in visual acuity (VA) [3]. In Canadian diabetics, the estimated prevalence of DME is 15.7% and the prevalence of visual impairment due to DME is 2.6% [4]. Vision loss is responsible for the highest direct healthcare expenditures of any major disease in Canada and the fourth highest indirect costs, primarily due to lost productivity [5]. Diabetic patients experience significant decrements in daily functioning and quality of life with increasing visual impairment [6]. The increasing prevalence of diabetes in Canada [7] and elsewhere will place more individuals at risk of DME.

The choice of treatment options for DME will depend on individual patient clinical characteristics [8]. While laser photocoagulation has been the mainstay for preventing vision loss, other options may include surgical vitrectomy and intravitreal corticosteroid injections. More recently, vascular endothelial growth factor (VEGF), an underlying cause of increased vascular permeability leading to macular edema, has been a therapeutic target in the treatment of DME. Early diagnosis and effective treatment of DME are essential to prevent visual impairment and avoid the consequential economic and societal impact of vision loss.

The impact of DME on the Canadian healthcare system is unknown. The most recent estimate of direct costs is based on implementing the 1998 practice guidelines for retinopathy and pre-2000 resource cost data [9]. The first year estimated cost for macular edema was $423 per patient and considered limited therapeutic (photocoagulation) and diagnostic procedure (color fundus photography and fluorescein angiography) options [9]. A US study, based on 2005 and earlier Medicare claims, compared direct medical costs of patients with incident DME versus diabetic patients with no retinal complications [10]. First year medical costs were $2,892 higher for patients with incident DME.

The objective of this study was to assess the direct and indirect DME-related costs of Canadian patients treated in clinical practice and measure their health-related quality of life. A further goal was to quantify costs and health-related quality of life by level of VA.

## 2. Materials and Methods

### 2.1. Study Design and Participants

This 6-month prospective observational study enrolled patients from September 28, 2010 to August 31, 2011. Retinal specialists and ophthalmologists at 16 sites in Canada recruited adult patients diagnosed with Type 1 or 2 diabetes and macular edema, either center involved macular edema or clinically significant macular edema (CSME) as defined by the Early Treatment of Diabetic Retinopathy Study (ETDRS) in one or both eyes. CSME was defined as the presence of one or more of the following: (1) thickening of retina at or within 500 *μ*m of the center of the macula; (2) hard exudates at or within 500 *μ*m of the center of the macula if associated with thickening of adjacent retina (but not residual hard exudates remaining after disappearance of retinal thickening); (3) a zone or zones of retinal thickening 1 disc area or larger, any part of which was within 1 disc diameter of the center of the macula. The ability to provide informed consent and complete office visits and telephone interviews was also required. Patients were excluded if they had intraocular surgery in the 3 months preceding the study, were currently enrolled or planned to enroll in a clinical trial, and/or had preexisting conditions that would adversely affect VA (e.g., active intraocular inflammation, age-related macular degeneration, cataract, choroidal neovascularization, glaucoma, macular hole, ocular histoplasmosis, pathologic myopia, retinal detachment, and retinal vascular occlusion).

The protocol, case report forms, and informed consent were approved by a central institutional review board and separate approvals were obtained at the facility level as needed. Each patient provided informed consent for study participation and confidentiality of all data was preserved.

### 2.2. Schedule of Assessments

Data were collected at the site at months 0 (baseline) and 6 (final) and during monthly telephone interviews with patients (months 1–6). Medical charts were abstracted at months 3 and 6.

### 2.3. Outcomes

Outcomes collected were VA, DME-related healthcare resource utilization, and health-related quality of life (HRQoL). Spectacle-corrected VA for both eyes was determined at baseline and final site visit using the Snellen chart with the exception of one site that used the ETDRS chart and, for these patients, the results were converted to Snellen units. If both eyes were diagnosed with DME, VA from the worst eye was used to classify patients into VA severity level. Direct medical DME-related healthcare resource utilization (treatment of DME and physician visits) was collected from the patient chart. Direct medical resources that were not expected to be available in the chart (other healthcare professionals, emergency room visits, and medical devices) and indirect costs including time loss from work were collected from patients during telephone interviews. Resources were assigned costs from the province of Ontario. If 2011 costs were not available, earlier costs were adjusted to 2010 $CAN [11]. Costs for consultations (provided by family doctors, ophthalmologists, optometrists, and retinal specialists), DME therapeutic (photocoagulation, retinal detachment surgery, surgical vitrectomy, and YAG laser), and diagnostic procedures (fluorescein angiography, optical coherence tomography) were obtained from the Ontario health insurance schedule of benefits and fees [12]. Travel expense of healthcare professionals was provided by patients at baseline and applied to each visit. Cost of fundus photography was obtained from an Ontario hospital. ER visit costs were computed from the sum of visit [13] and ER physician fees [12]. The cost of ambulance service was based on yearly government expenditures divided by the annual number of services provided. Fees for nurse consultation were obtained from the Ontario Nurses Association. Canadian National Institute for the Blind (CNIB) per visit fees were computed from the per patient cost of government sponsored training and rehabilitation programs divided by an estimated 15 visits [14]. Medical device cost data (glasses and frames, magnifiers, lamps, and walking aids) were provided by local retail outlets. Time missed from work was valued at the Canadian average industrial wage rate [15].

The unit cost of corticosteroids (assumed to be the cost of triamcinolone since it was the best available price on the Ontario formulary) [16], medications to treat complications of DME treatment (brimonidine/timolol and moxifloxacin) [16], ranibizumab (Lucentis) [16] and bevacizumab (Avastin) included an $8 dispensing fee, 8% markup, and, for injectable drugs, a $210 physician fee. The unit cost of bevacizumab was obtained from a hospital-based pharmacy. Because the names of anti-VEGF medications were not collected, it was assumed that 70% of the patients received bevacizumab ($70.19 including markup) and 30% received ranibizumab ($1,701 including markup). A larger proportion of patients were assumed to have received bevacizumab because ranibizumab for the treatment of DME was not yet approved for reimbursement in most provinces. Further, halfway through the study, the listing status of ranibizumab was changed to a limited use product for the treatment of wet age-related macular degeneration only on the Ontario Drug Benefit Formulary and a large proportion of the patients were from Ontario. Thus, the unit cost of anti-VEGF medication (including dispensing, physician fees, and $4 for ofloxacin ophthalmic (Ocuflox)) derived from the assumed 70 : 30 proportion of patients was $781.27.

HRQoL was assessed at baseline and months 3 and 6 with the National Eye Institute Visual Functioning Questionnaire-25 item (NEI VFQ-25) [17] and EuroQol 5 dimensions (EQ-5D) [18] instruments. The NEI VFQ-25 contains 11 subscales including vision specific subscales and a single item general health rating with higher scores representing better function. Scores were computed for the composite, each subscale, quality of vision [19] (mean of general vision, near activities, distance activities, peripheral vision, and colour vision subscales) and vision-related quality of life (mean of driving, ocular pain, role difficulties, dependency, social functioning, and mental health subscales) [19]. The EQ-5D is a generic preference instrument with higher scores representing better health status. The utility score represents health status according to 5 dimensions: mobility, self-care, usual activities, pain/discomfort, and anxiety/depression. Overall health state is measured on a 0–100 visual analogue scale (VAS).

### 2.4. Analysis

Costs and HRQoL were summarized by VA at baseline using a classification scheme for age-related macular edema (AMD): normal/mild (VA 20/10 to >20/80); moderate (VA ≤ 20/80 to >20/200); severe (VA ≤ 20/200) [14]. Patients who dropped out of the study at 3 months or earlier were excluded, and costs for patients who had at least one interview after month 3 were standardized to 6-month costs.

## 3. Results

### 3.1. Patient Sample and Baseline Characteristics

A total of 145 patients were enrolled across 16 sites from 6 provinces: 43 (29.7%) by ophthalmologists and 102 (70.3%) by retinal specialists. Twenty-one patients dropped out before month 6, 135 patients had sufficient data to compute 6-month costs, and 129 patients completed the NEI VFQ-25 and EQ-5D questionnaires at 6 months. Demographic and clinical characteristics of patients at baseline are shown in Table 1. The majority of patients had Type 2 diabetes (81%) and nonproliferative retinopathy (73%). Sixty-one percent of eyes (*n* = 249) were diagnosed with focal DME. The percentage of patients in each VA category at baseline was normal vision/mild loss, 63%; moderate loss, 10%; severe loss/nearly blind, 26%. The mean VA was 20/60 or logMAR0.49.

### 3.2. DME-Related Healthcare Resource Utilization and Costs

Resource utilization during the 6-month study, the unit cost of each resource, the number of patients who used each resource, and the mean number per patient (over all patients) are listed in Table 2. Anti-VEGF injections were received by 29% of patients. The mean number of injections across all patients was 1.31. The mean number of injections across the 39 patients who received anti-VEGF was 4.5 per patient (data not shown). Laser photocoagulation was the most common therapy (57% of patients) and optical coherence tomography used to monitor center involved macular edema was the most common procedure (58% of patients). Most patients had retinal specialist visits (79%), which is reflective of the greater proportion of retinal specialists who enrolled patients. Thirty-two percent of patients used a medical device. Thirteen percent of patients and 11% of caregivers had lost time from work due to DME symptoms, treatment, or sight-related accidents. Although this was half a day per patient across all patients, out of the 49 patients (34%) who worked full-time or part-time, the mean number of days missed per patient was 1.4 days (data not shown).

**Figure 1:**
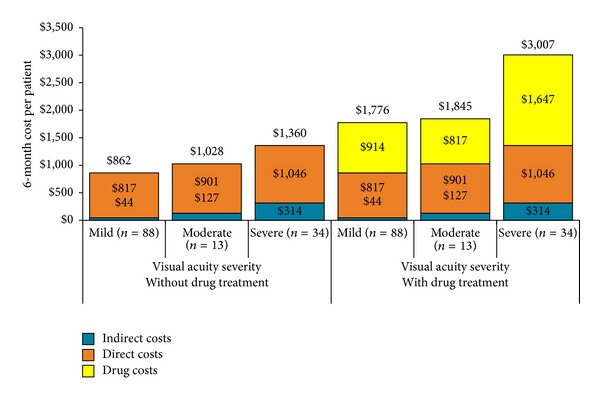
Mean 6-month DME-related costs per patient by visual acuity severity: with and without drug costs.

Mean total DME-related costs over 6 months were $2,092/patient (95% CI: $1,694, $2,490) (Table 3). The primary cost driver was anti-VEGF, accounting for 49% of total costs, followed by healthcare professional visits (14%) and laser photocoagulation and surgical vitrectomy (12%). Costs by VA level are shown in Figure 1. Because the names of the anti-VEGF drugs were not collected and assumptions were made regarding the proportion of patients receiving each of the 2 anti-VEGF treatments, costs are reported with and without drug treatment costs. Excluding drug costs, mean total costs increased with increasing VA severity, from $862 for patients with normal/mild vision loss to $1,360 for severe VA loss and the pattern of increasing costs was evident for both direct (medical costs) and indirect costs (time loss from work). Mean total costs including drug treatment were highest in the severe VA category ($3,007).

### 3.3. Health-Related Quality of Life

**Figure 2:**
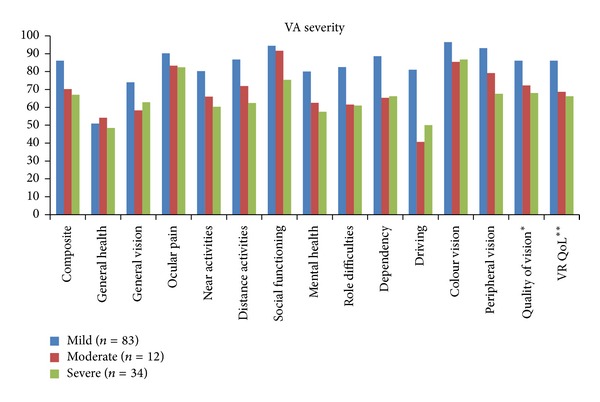
Mean NEI VFQ-25 scores at 6 months by visual acuity level. *Mean of general vision, near activities, distance activities, peripheral vision, and colour vision subscales. **Mean of driving, ocular pain, role difficulties, dependency, social functioning, and mental health subscales. VR Qol: vision-related quality of life.

Mean NEI VFQ-25 scores at 6 months for all patients and by the VA severity category are provided in Table 4 and Figure 2, respectively. Patients with normal/mild vision loss had the highest scores for the composite scale, the two summary scales, and all subscales, with the exception of the general health subscale. A pattern of decreasing score with increasing VA severity (using 1 point score difference between VA severity levels) was evident for the composite, near and distance activities, social functioning, mental health, peripheral vision, quality of vision, and vision-related quality of life.

**Figure 3:**
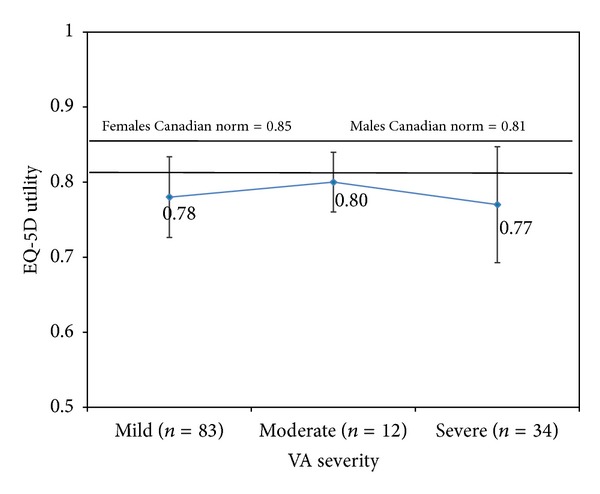
Mean EQ-5D utility scores and 95% confidence intervals at 6 months by visual acuity severity.

Mean EQ-5D scores at 6 months for all patients are in Table 4; scores for each VA severity category are shown in Figures 3(utility) and 4(VAS). For all VA categories, both utility and VAS scores were lower than Canadian norms; patients with moderate vision loss had the highest utility and VAS scores and patients with severe loss had the lowest scores, with the difference between the severity levels being larger for the VAS results.

## 4. Discussion

We prospectively examined 6-month DME-related healthcare utilization, cost, and HRQoL of patients with DME in Canada. To our knowledge, this is the first study to directly capture the burden of DME in Canadian patients.

Over 6 months, optical coherence tomography was the most common diagnostic procedure (58% of patients), followed by fluorescein angiography (16%) and fundus photography (13%). In a retrospective US study of Medicare patients with incident DME, Shea et al. reported that approximately 3% of patients underwent optical coherence tomography in 2000 but increased to more than 40% of patients by 2004 [10]. The improved sensitivity of optical coherence tomography versus stereoscopic slit-lamp examination for the detection of center involved macular thickening was reported in 2004 [20] and our results likely reflect an increasing clinical adoption of this practice. Laser photocoagulation was the most prominent therapy (57% of patients) in this study and in the report by Shea et al. (38%); laser photocoagulation is widely recognized as the standard of care to reduce vision loss in patients with DME and our results reflect this practice. This study demonstrates the emerging use of anti-VEGF agents for DME treatment, with 29% of patients receiving an anti-VEGF injection and 11% receiving steroid injections for center involved macular edema. At the time of the study, there were no approved anti-VEGF agents for the treatment of DME; however, anti-VEGF treatment was approved for other indications and was used to treat DME patients. Anti-VEGF treatment has been shown to not only maintain vision similar to laser therapy but also improve vision especially in persons with center involved macular edema for whom there was limited treatment options prior to the introduction of anti-VEGF therapy, which may explain its emerging use in this study [21, 22].

From a societal perspective, total mean DME-related costs per patient over 6 months were $2,092 or annualized to $4,184. The first year annual cost of DME-related healthcare resource utilization in Canada has been previously estimated at $423 ($CAN 2000) [9]. However, this estimate is not based on real world practice, but rather implementation of Canadian practice guidelines published in 1998, and included only costs associated with physician visits, ophthalmology consults, and diagnostic (i.e., color fundus photography, fluorescein angiography) and therapeutic procedures (photocoagulation) in use at the time of the study. Two retrospective US studies, based on claims data from 2004 and earlier, examined the incremental costs of retinal complications in patients with diabetes. The annual incremental cost of DME, including lost work productivity, was $12,244 ($US 2005) [23] and the annual incremental cost of incident DME, based on direct medical costs, was $2,892 ($11,290 in the DME groups versus $8,398 in the control group) ($US 2002) [10]. In a cross-sectional study based on medical chart review and patient interviews, Cruess and colleagues examined the direct vision-related medical costs of Canadian patients with bilateral neovascular AMD [14]. Total mean direct vision-related annual costs were $6,314 ($CAN 2005); excluding direct medical treatment, costs were $1,007 which is similar to our annualized costs of $975 excluding treatment costs [14]. The Cruess study reported an increase in overall costs as VA severity worsened but a reduction in direct vision-related medical costs as VA severity worsened although not statistically significant. This can be compared with the current study which found an increase in costs as severity worsened. Differences in study methodology, patient characteristics (including severity of retinal disease and other diabetic complications), and the available treatments during the course of each study likely contributed to the variation in estimated costs. However, all studies underscore the significant impact of retinal disease on healthcare costs.

As VA severity worsened, a trend for decreasing quality of life using the NEI VFQ-25 was evident; however the scores between patients with moderate and severe VA did not always differ. The small sample size in the moderate VA severity (*n* = 12) group may explain some of the findings for this group. According to one analysis, the clinically important difference for the composite score is 4 points, which is associated with a 15-letter change, and 3 points which is associated with a 10-letter change; for the subscales, the clinically important differences are approximately 2 to 6 points for a 15-letter change and 1 to 4 points for a 10-letter change [24]. We did observe a difference of 3 or more points in the composite score across the 3 VA severity levels. Comparing the difference between the normal/mild vision loss category and the moderate and severe categories, we observed decrements of at least 4 points for both summary scores and all subscales except general health and, at the moderate VA level, social functioning. It is not surprising that general health scores do not differ by VA level since it relates to more broad health status than the other subscales which focus on quality of life related to the patients' eyesight.

The mean composite score ranged from 86.0 for normal/mild vision loss to 67.0 for severe vision loss which is similar to the range of 84.0 (normal vision) to 65.5 (severe vision loss) reported for diabetic retinopathy patients [6]. However, our quality of vision, vision-related quality of life, and subscales results were higher than those observed in a US study of Type 2 diabetes patients with DME [19]. Patient age (64) and duration of diabetes (16 years) were similar to our patient sample; however it is not possible to determine if the differences could be attributable to VA because we determined VA based on the worst-seeing eye (logMAR0.46) and the earlier study [19] was based on the best-seeing eye (logMAR0.35). Our results are also based on a larger sample size: 129 patients versus 33 patients. A European study [25] of 401 patients with neovascular AMD, including 67 patients in Canada [14], also identified a pattern of declining NEI VFQ-25 composite scores with increasing visual loss. Although the mean composite score for our patients with DME was lower than the control group for the AMD study by 10 points [25], they were approximately 30 points higher than scores for all AMD patients [25] and the subset of Canadian patients [14]. In addition to differences in disease etiology, mean patient age was 64 years in this study versus 78 years for AMD patients [25] and mean VA in the best-seeing eye was worse (logMAR0.60) [25] than our VA scores based on the worse-seeing eye (logMAR0.35). These differences provide a rationale for the higher NEI VFQ-25 scores we observed.

The EQ-5D utility and VAS scores did not demonstrate a pattern of decline with increasing visual loss, although mean utility scores were lower than Canadian norms (0.85 females and 0.81 males) at each VA category. The mean utility score of 0.78 for our DME patients was higher than the score reported for AMD patients (0.65) [25], similar to the elderly control population without AMD (0.75) [25], and lower than patients with diabetes but no retinopathy (0.83) [26]. Neither study identified a consistent decline in utility scores with increasing vision loss in patients with AMD or diabetic retinopathy. Fenwick and colleagues modeled the association between vision impairment and utility scores in patients with diabetic retinopathy and/or DME in Australia and also found no significant relationship [27]. However, one study in diabetic retinopathy patients did note declining utility scores using the time trade off technique with increasing visual impairment in the best-seeing eye [28]. The EQ-5D is a generic preference instrument and does not contain vision-related functioning elements. Our results, coupled with these other studies, suggest that the EQ-5D is not sensitive to changes in functioning and HRQoL associated with visual impairment.

A strength of this study was that patients were followed prospectively and it was possible to measure VA during the study and assign patients to severity of VA to determine if costs and HRQOL differed across VA severity. A limitation of the classification of VA severity was that it was based on the worst-seeing eye to correlate with costs; however the correlation with HRQoL may have been stronger if the classification of severity was based on the best-seeing eye. Another strength is that healthcare resources were collected from the physician chart and also from the patient for resources not found in the chart. Costs included indirect and direct costs, and overhead costs were included where available. The study had other limitations. Our costs results are based on unit costs reported for services provided in the province of Ontario but may vary within and beyond Ontario. Anti-VEGF agents represented the largest component of DME-related costs and there was a substantial difference in unit cost of the agents (bevacizumab and ranibizumab) available at the time of the study. It was assumed that 70% of the patients received bevacizumab and 30% received ranibizumab for a mean anti-VEGF injection cost per patient of $1,024.33 and a mean total cost of $2,092.18 per patient. Irrespective of assumptions, these results demonstrate the substantial economic burden of DME in Canada. The study also lacked a control group and we grouped patients according to previously reported levels of VA; however, the sample size differed among these groups which precluded formal statistical evaluation of the effect of visual impairment on costs and HRQoL. Yet, our results are based on patients diagnosed and treated for DME in real world clinical practice and, thus, are likely to be more broadly generalizable than results of clinical trials with more narrow inclusion criteria.

DME represents a considerable burden to the Canadian healthcare system and patients. DME-related costs averaged $2,092 over 6 months, and, excluding drug costs, increased as VA severity increased. Drug costs were highest in patients with severe VA. The burden of DME also included a reduction in functional ability and HRQoL, as measured by the NEI VFQ-25 and EQ-5D. Although the EQ-5D did not demonstrate a pattern of decline with increasing visual loss, functional ability measured by the NEI VFQ-25 declined at higher levels of visual impairment. Prevention of avoidable vision loss is the primary goal of DME treatment. EQ5D might not be sensitive enough to differentiate patients with different VA levels. Measurements from disease specific questionnaires might be able to measure all the aspects of visual function affected by visual impairment compared with EQ-5D, a generic instrument, and this should be studied further. Early diagnosis and effective treatment will improve visual outcomes, preserve quality of life, and reduce the long-term burden of illness.

## Figures and Tables

**Figure 4:**
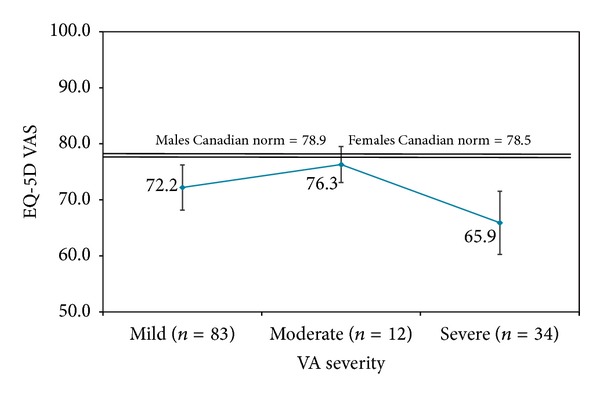
Mean EQ-5D visual analogue scale (VAS) scores and 95% confidence intervals at 6 months by visual acuity severity.

**Table 1:** Baseline patient characteristics.

Characteristic*	Patients (*N* = 145)
Males, *n* (%)	76 (52.4)
Age (years),	
Mean (SD)	63.7 (10.7)
Min, median, max	30, 65, 86
Diabetes, *n* (%)	
Type 1	25 (17.2)
Type 2	118 (81.4)
Missing	2 (0.8)
Diabetes duration (years), mean (SD)	
Type 1	26.8 (13.3)
Type 2	16.2 (9.0)
HbA1c, mean (SD) (*n* = 79)	7.7 (2.0)
Type of diabetic retinopathy, *n* (%) (*n* = 138)	
Proliferative	37 (25.5)
Nonproliferative	106 (73.1)
Proliferative and nonproliferative^†^	1 (0.7)
Missing	1 (0.7)
Type of DME, # of eyes, *n* (%) (*n* = 249)	
Focal	152 (61.0)
Diffuse	91 (36.5)
Focal and diffuse^‡^	4 (1.6)
Missing	2 (0.8)
CSME diagnosis, *n* (%)	
Both eyes	104 (71.7)
Right eye only	16 (11.0)
Left eye only	25 (17.2)
VA severity, *n* (%)^§^	
Normal/mild (VA 20/10 to >20/80)	92 (63.4)
Moderate (VA ≤20/80 to >20/200)	15 (10.4)
Severe/nearly blind (VA ≤20/200)	38 (26.2)
Drug plan^¶^, *n* (%)	
Government plan	65 (44.88)
Employer/private plan	73 (50.3)
No plan	9 (6.2)
Missing	4 (2.8)

*All characteristics were extracted from medical charts with the exception of HbA1c and drug plan which were patient-reported.

^†^One patient reported “proliferative” in left eye and “nonproliferative” in right eye.

^‡^Two patients reported both focal and diffuse in each eye.

^§^Severity is based on VA at baseline in the worst eyes if both eyes were diagnosed with DME.

^¶^Patients may report more than one plan.

CSME: clinically significant macular edema; DME: diabetic macular edema; HbA1c: hemoglobin A1c.

**Table 2:** DME-related health resource utilization over 6 months.

Resource	Cost, $CAN 2010/2011	Patients (*N* = 135)		Mean # per patient across all patients (*N* = 135)
		n*	%	
Drug treatment				
Steroid injection	223.15/injection	15	11.1	0.24
Anti-VEGF injection	781.27/injection	39	28.9	1.31
Treatment of adverse events^†^				
Brimonidine/timolol^‡^	1.44/day	1	0.7	
Moxifloxacin^§^	6.29/day	11	8.2	
Therapy				
Laser photocoagulation	187.25/procedure	77	57.0	1.14
Surgical vitrectomy/epiretinal membrane peel	892.03/procedure	8	5.9	0.06
Procedures				
Fluorescein angiography	23.20/procedure	22	16.3	0.16
Optical coherence tomography	70.00/procedure	78	57.8	1.21
Fundus photography	35.00/procedure	17	12.6	0.33
Healthcare professionals				
Ophthalmologist	71.30/initial visit; 50.00/subsequent visit	51	37.8	1.33
Retinal specialist	71.30/initial visit; 50.00/subsequent visit	106	78.5	3.11
Optometrist	47.00/visit	25	18.5	0.26
Family doctor	33.10/visit	19	14.1	0.19
Nurse	35.90/visit	1	0.7	0.01
CNIB	135.54/visit	1	0.7	0.01
Home care	53.71/visit	2	1.5	0.23
ER visit by ambulance	876.29/visit	1	0.7	0.01
Use of medical device	10.80–350.00/device^¶^	43	31.9	NA
Patient missed days from work due to DME	185.40/day	18	12.7	0.48
Caregiver missed days from work due to patient's DME	185.40/day	15	10.6	0.16

*Patients may have received more than one therapy.

^†^Medications prescribed for the prevention of complications such as medications given as prophylaxis for endophthalmitis.

^‡^Mean duration of treatment: 90 days.

^§^Mean duration of treatment: 13.2 days.

^¶^Costs varied depending on device, for example, $10.80 for safety glasses and $350 for new glasses prescription charge.

CNIB: Canadian National Institute for the Blind; DME: diabetic macular edema; ER: emergency room; NA: not applicable; VEGF: vascular endothelial growth factor.

**Table 3:** Mean 6-month DME-related cost per patient.

	Item	Mean (*N* = 135)	SD	% of Total Cost
Direct costs	Drug treatment			
	Intravitreal steroid injections	$53	$169	3%
	Anti-VEGF	$1,024	$2,033	49%
	Medications for adverse effects of DME treatment	$12	$52	<1%
	Therapies (laser photocoagulation, surgical vitrectomy)	$260	$298	12%
	Procedures	$100	$132	5%
	Healthcare professionals	$295	$191	14%
	Patient travel to healthcare professional	$135	$173	6%
	Medical devices	$85	$162	4%
	ER visits	$3	$31	<1%
	Ambulance	$4	$44	<1%

Indirect costs	Time loss from work patient	$90	$720	4%
	Time loss from work caregiver	$30	$117	1%

Total		$2,092	$2,339	

ER: emergency room; VEGF: vascular endothelial growth factor; SD: standard deviation.

**Table 4:** Mean NEI VFQ-25 and EQ-5D scores at 6 months for all patients.

Instrument	Mean (*N* = 129)	SD
NEI VFQ-25*		
Composite	79.6	18.7
Quality of vision^†^	80.0	17.5
Vision-related quality of life^‡^	79.2	21.4
Subscales		
General health	50.6	22.0
General vision	69.6	16.4
Ocular pain	87.5	17.8
Near activity	73.7	25.5
Distance activity	78.9	23.4
Social functioning	89.1	19.8
Mental health	72.5	28.5
Role difficulties	74.9	29.3
Dependency	80.6	30.5
Driving	69.9	30.7
Color vision	92.8	16.6
Peripheral vision	85.1	24.5
EQ-5D		
Utility^§^	0.78	0.23
VAS^¶^	71.0	17.7

*All scores ranged from 0 to 100. Higher scores represent better functioning.

^†^Quality of vision is defined as the mean score of the following subscales: general vision, near activities, distance activities, peripheral vision, and color vision.

^‡^Vision-related quality of life is defined as the mean score of the following subscales: driving, ocular pain, role difficulties, dependency, social functioning, and mental health.

^§^The higher the score the better the quality of life, where 0 represents death and 1 represents perfect health.

^¶^Scores range from 0 to 100, where 0 represents the worst imaginable health state and 100 represents the best imaginable health state.

EQ-5D: Euroqol 5 dimensions; NEI VFQ-25: National Eye Institute Visual Functioning Questionnaire-25 item; VAS: visual analogue scale.
